# High-Throughput Phytochemical Unscrambling of Flowers Originating from *Astragalus membranaceus* (Fisch.) Bge. var. *mongholicus* (Bge.) P. K. Hsiao and *Astragalus membranaceus* (Fisch.) Bug. by Applying the Intagretive Plant Metabolomics Method Using UHPLC−Q−TOF−MS/MS

**DOI:** 10.3390/molecules28166115

**Published:** 2023-08-18

**Authors:** Qi Liu, Jinghui Li, Meiling Gu, Wanying Kong, Zhao Lin, Jialin Mao, Meng Zhang, Liyan Jiang, Can Liu, Yumei Wang, Jicheng Liu

**Affiliations:** 1 The Research Institute of Medicine and Pharmacy, Qiqihar Medical University, Bukui Street 333, Qiqihar 161006, China; liuqi_hlj@163.com (Q.L.); ljh20120525@126.com (J.L.); 13969409385@163.com (M.G.); kong0604@126.com (W.K.); 13244328706@163.com (Z.L.); maojialin1026@163.com (J.M.); 18098728279@163.com (M.Z.); qjiangliyan@163.com (L.J.); lc1349847764@163.com (C.L.); 2 The Research Institute of Astragalus Industry, Qiqihar Academy of Medical Sciences, Qiqihar Medical University, Bukui Street 333, Qiqihar 161006, China

**Keywords:** *Astragalus membranaceus* (Fisch.) Bge. var. *mongholicus* (Bge.) P. K. Hsiao flower, *Astragalus membranaceus* (Fisch.) Bug. flower, metabolomics, UHPLC−Q−TOF−MS/MS, markers

## Abstract

*Astragalus membranaceus* (Fisch.) Bge. var. *mongholicus* (Bge.) P. K. Hsiao (MO) and *Astragalus membranaceus* (Fisch.) Bug. (ME) are two primary sources of the *Astragalus* herb*,* also known as “Huangqi” in China, which is widely applied to treat hypertension, glomerulonephritis, ischemic heart disease, and diabetes mellitus. As two different sources of the *Astragalus* herb, the chemical profiles of MO and ME may be different. Previous studies showed abundant differences in chemical composition between MO and ME. Therefore, the by-products of MO and ME, such as *Astragalus membranaceus* (Fisch.) Bge. var. *mongholicus* (Bge.) P. K. Hsiao flower (MOF) and *Astragalus membranaceus* (Fisch.) Bug. flower (MEF), may have different phytochemical profiles. In this paper, a metabolomics method combined with ultra-high-performance liquid chromatography and electrospray ionization/quadrupole time-of-flight mass spectrometry (UHPLC−Q−TOF−MS/MS) was employed to analyze the components of MOF and MEF. Consequently, the results of principal component analysis (PCA) showed that MOF and MEF could be separated clearly. In total, 31 chemical markers differentiating MOF and MEF were successfully identified, including 22 flavonoids, 8 isoflavones and 1 benzopyran. Among them, the contents of 18 components, including Calycosin, Cyanidin-3-O-glucoside, Quercetin, Rutin, Kaempferol, Formononetin, Isomucronulatol and Prim-O-glucosylcimifugin in MEF, were significantly higher than in MOF. In turn, the contents of another 13 components, covering Biochanin A, Tectoridin, Isomucronulatol-7-O-glucoside, Liquiritin, Rhamnetin, etc., were lower in the MEF group than that in the MOF group. It is worth noting that flavonoids, especially flavonoid glycosides, were the primary active chemical ingredients in MOF and MEF. The 18 ingredients in MEF with a higher level carried out diverse activities, like anti-oxidant, anti-inflammatory, anti-bacterial and anti-tumor activities, which led us to speculate that MEF may have greater pharmacological effects and potential development prospects than MOF. The present results displayed that the contents of ingredients in the two different species of plants were radically different, and there was significant uniqueness to the components of MOF and MEF. Our study not only provides helpful chemical information for further quality assessment and active mechanism research of MOF and MEF but also offers scientific support for the resource utilization of MOF and MEF.

## 1. Introduction

*Astragalus membranaceus* (Fisch.) Bge. var. *mongholicus* (Bge.) P. K. Hsiao (locally known as “menggu huangqi”, MO) and *Astragalus membranaceus* (Fisch.) Bug. (locally known as “mojia huangqi”, ME), two perennial leguminous plants with wide distribution, belong to the same traditional Chinese medicine (TCM) named “Huangqi” in *Chinese Pharmacopoeia* (2020 edition). Generally, MO and ME are widely applied for immunity improvement [1], cardiotonic enhancement [2], anti-hypertension [3], anti-cancer [4], anti-virus [5] and anti-diabetes [6,7,8] treatments. Our previous study proved that the chemical components of MO and ME were obviously different [9]. Due to different origins of MO and ME, their by-products may also express disparate chemical labels. *Astragalus membranaceus* (Fisch.) Bge. var. *mongholicus* (Bge.) P. K. Hsiao flower (MOF) and *Astragalus membranaceus* (Fisch.) Bug. flower (MEF) are two outcomes of MO and ME. Their extracts and chemical substances are usually used as substitutions for medicine, health products, scented tea, food and even cosmetics in China [10]. In recent years, on account of the utilization of above-ground resources, MOF and MEF have been paid much more attention than in the past. Studies showed that the chemical components of different organs from “Huangqi” covered multiple ingredients such as flavonoids, saponins, polysaccharides, vitamins, amino acids and elements [11,12], and there were some differences between the by-product part and the root part [13,14]. However, before now, overall chemical research on MOF and MEF was rarely reported, and the chemical differences between MOF and MEF have not been exploited yet.

For medicinal plants, the production and distribution of secondary metabolites is usually unique during different growth stages and among diverse species, organs and tissues. Phytochemical unscrambling is the primary means of exploring the potential value of MOF and MEF. However, before now, the secondary metabolites of MOF and MEF have been rarely reported [15]. In recent years, with the rapid development of modern analytical means, the combination of high-throughput LC-MS and metabolomics has been widely used in phytochemical analysis of medicinal plants [16,17,18], and it may be a powerful tool for analyzing the components of MOF and MEF.

In the present paper, a full-scale metabolomics method based on ultra-high-performance liquid chromatography coupled with electrospray ionization/quadrupole time-of-flight mass spectrometry (UHPLC−Q−TOF−MS/MS) technology is employed to understand the chemical discrimination of MOF and MEF. Our study provides full-scale phytochemical information for further quality assurance and a furnished and rigorous basis for the pharmacological study of MOF and MEF. Moreover, the results reveal that the plant metabolomics method is powerful for discriminating between the diverse sources of traditional Chinese medicine. Our presented data provide a comprehensive component basis for far-reaching effective utilization of MOF and MEF, which is beneficial for the exploitation of non-medicinal plant parts for “Huangqi” in China. 

## 2. Results and Discussion

### 2.1. Conditions for UHPLC−Q−TOF−MS/MS Platform

**Figure 1:**
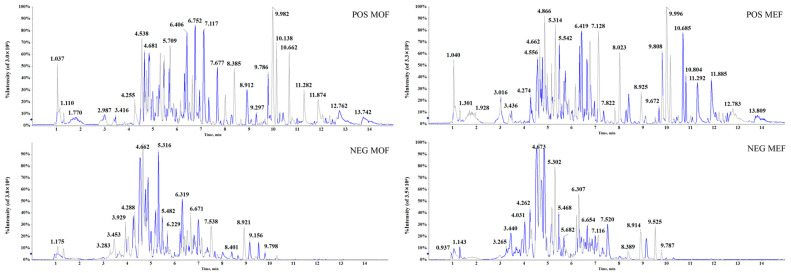
The base peak chromatograms of MOF and MEF under positive ion mode and negative ion mode by UHPLC−Q−TOF−MS/MS.

The conditions of the UHPLC−Q−TOF−MS/MS platform were vital for plant metabolomics analysis. In this analysis, two separation columns, ACQUITY™ BEH C_18_ column (100 mm × 2.1 mm i.d., 1.8 µm) and ACQUITY™ UPLC HSS T_3_ column (100 mm × 2.1 mm i.d., 1.8 µm), were investigated, and the latter produced preferable results with a much more graceful shape and a larger quantity and greater intensity of peaks. Additionally, various kinds of constituents with unequal polarity could be separated using an appropriate mobile system. Formic acid was an ideal acid for the mobile phase in LC-MS/MS. During the analysis of TCM, whether the ingredients were acidic or alkaline, the mobile phase with formic acid could easily achieve better peak shapes and significantly improve the column’s effect. In the aforementioned separation process, 0.1% formic acid in the aqueous phase (water) and organic phase (acetonitrile) showed acceptable data. Furthermore, a flow rate of 0.4 mL/min and injection volume of 3 μL exhibited a nice resolution of chromatographic peaks. MS arguments covering ISVF, ESI temperature and nitrogen pressures of GS1, GS2 and CUR were the recommended values, which could result in the neat separation and response of multifold peaks in TCM. For favorable acquisition efficiency, the IDA acquisition method with high performance was employed to simultaneously obtain MS and MS/MS data. The previous ten ions in the accumulation interval stage appeared to be significant ions matching with the IDA. The dynamic background subtraction method was applied in order to distinguish the background and interrelated fragments. Under the aforementioned conditions of UHPLC−Q−TOF−MS/MS, high-quality base peak chromatograms (Figure 1) with favorable separation and response under the positive ion mode (POS) and negative ion mode (NEG) were obtained.

### 2.2. Multivariate Process and Statistical Data Analysis for Metabolomics Profiling

**Figure 2:**
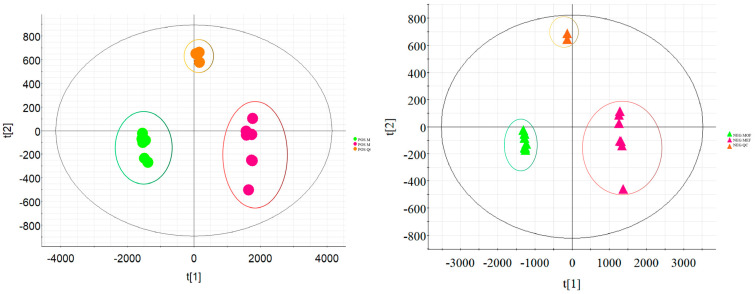
The PCA plots between MOF and MEF under positive ion mode and negative ion mode by UHPLC−Q−TOF−MS/MS approach.

After the raw metabolomics data were processed, screened ions were further subjected to several statistical analyses. During data acquisition, three QC samples were stayed closely together on the principal component analysis (PCA) score plots, indicating that the stability, quality and reproducibility of UHPLC−Q−TOF−MS/MS instrument were favorable. Next, multivariate orthogonal partial least squares-discriminant analysis (OPLS-DA) was conducted. For the metabolomics study, large samples undoubtedly provided richer information for research; however, the workload of these data was overtly increased. More importantly, there may be numerous correlations among variables in most cases. Separately analyzing each indicator was unreasonable and unilateral. The use of comprehensive multivariate data based on overall information was much more realistic. The obtained data were processed using a non-supervised PCA and supervised OPLS-DA. PCA, which can locate vital information among complex data clusters, was widely applied in the reduction and separation of variables among different classes. In the present paper, PCA maps are shown in Figure 2. The MOF and MEF were overtly distributed into two distinct regions, indicating that there were evident differences between the MOF group and the MEF group. 

**Figure 3:**
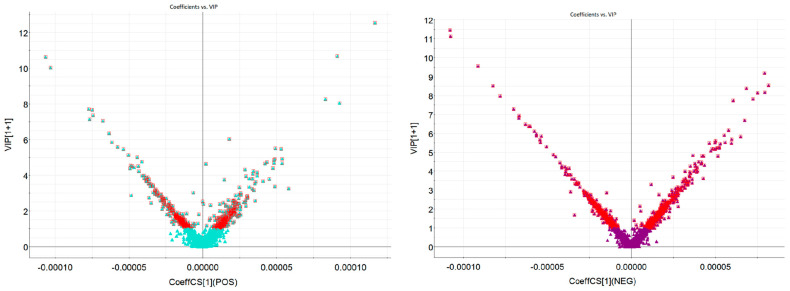
The VIP maps of ions between MOF and MEF under positive ion mode and negative ion mode by UHPLC−Q−TOF−MS/MS. (Blue-green square: scrrened ions whose VIP ≥ 1.0 under positive ion mode; purple triangle: scrrened ions whose VIP ≥ 1.0 under negative ion mode.)

In order to screen the chemical metabolites of the MOF group and MEF group, the variable importance in the projection (VIP) maps were established to appraise the contribution value of the filtered variables in the OPLS-DA model. Ions whose values were greater than or equal to 1.0 could statistically represent the characteristics of differential ions. The VIP plot maps are shown in Figure 3. As displayed in Figure 3, screened ions (VIP ≥ 1.0) were deemed to be the most remarkable chemical markers between the MOF group and MEF group. Under applicable VIP values, the nominated ions were successfully screened out with high reliability for further identification procedures. 

### 2.3. Rapid Identification of Chemical Metabolites between MOF Group and MEF Group

**Figure 4:**
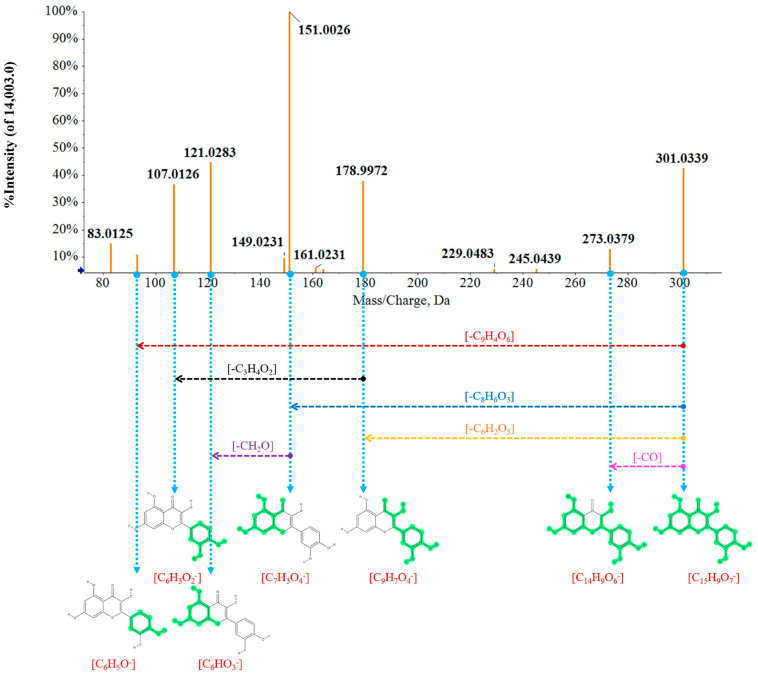
The detailed fragments and cracking processes of Quercetin in negative ion mode by UHPLC−Q−TOF−MS/MS. (Gray parts in structures: the possible loss fragments during the cracking processes. Green parts in structures: the possible remaining strutures during the cracking processes).

Based on the multivariate data results of metabolomics, the screened differential candidates between MOF and MEF were identified in order. For example, the identification processes of a representative compound named Quercetin was described in detail. The precursor ion was detected in the QC sample under the NEG ion mode, the retention time was 5.21 min and the [M − H]^−^ was 301.0339 Da. The molecular formula was calculated as C_15_H_10_O_7_ according to the rule of elemental composition and isotope abundance. The degree of unsaturation was 12, suggesting that the ion may contain one or two ring structures. Furthermore, the fragments in the MS/MS spectrogram included *m*/*z* 273, *m*/*z* 179, *m*/*z* 151, *m*/*z* 121, *m*/*z* 107 and *m*/*z* 93, speculating that the fragments might be C_14_H_9_O_6_^−^, C_9_H_7_O_4_^−^, C_7_H_3_O_4_^−^, C_6_HO_3_^−^, C_6_HO_2_^−^ and C_6_H_5_O_3_^−^, according to experience. At last, in order to confirm the inference results, the data were matched with the fragment database and finally accurately identified as Quercetin. The possible fragmentation processes are displayed in detail in Figure 4. 

In line with the identification procedures, 31 chemical metabolites (12 ions in the POS ion mode and 19 ions in the NEG ion mode) were provisionally identified. Comprehensive information including name, retention time, molecular weight, molecular formula, adducts and mass error is exhibited in Table 1, covering Liquiritin (**C1**), Isomucronulatol-7-O-glucoside (**C2**), Herbacetin-3,8-diglucopyranoside (**C3**), 4′,5-Dihydroxy-3′,6,7-trimethoxyflavone (**C4**), Luteolin-7,3′-di-O-glucoside (**C5**), Hovetrichoside C (**C6**), Quercetin 3,4′-diglucoside (**C7**), 3-galactopyranoside (**C8**), Rutin (**C9**), Kaempferol-7-O-neohesperidoside (**C10**), Kaempferol (**C11**), Kaempferol 3-ss-D-galactoside (**C12**), 6″-O-Malonylisoquercitrin (**C13**), 6″-O-Malonylgenistin (**C14**), Cyanidin-3-O-glucoside (**C15**), 3-O-glucoside (**C16**), Kaempferol Tamarixetin (**C17**), Prim-O-glucosylcimifugin (**C18**), Rhamnetin (**C19**), 3-O-(3″,4″-di-O-acetyl-α-L-rhamnopyranoside) (**C20**), 6″-O-Malonylglycitin (**C21**), Quercetin (**C22**), Quercetagetin (**C23**), Calycosin (**C24**), 3,5,7-Trihydroxy-4′-methoxyflavone (**C25**), Diosmetin-7-O-β-D-glucopyranoside (**C26**), Tectoridin (**C27**), Luteolin 7-methyl ether (**C28**), Formononetin (**C29**), Isomucronulatol (**C30**) and Biochanin A (**C31**). To our surprise, it was worth noting that the retention times of identified constituents were mostly gathered in the range of 3–6 min, indicating that the polarity of these compounds were in close proximity. Commonly, for the chemical analysis of TCM, components with the same class may have similar polarity. The present results offered an important reference for further extraction solvent selection in the optimization of preparation.

### 2.4. Comparison of Intensity of Flavonoid Metabolites between MOF and MEF

**Figure 5:**
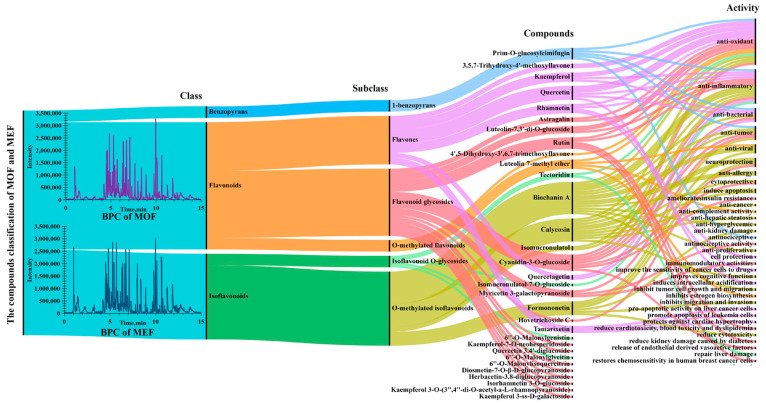
The refined classes and related activities of identified ingredients between MOF and MEF.

In order to explore the activities of different markers in depth, a Sankey map combining classes and activities of chemical ingredients was drawn and is displayed in Figure 5. As seen in Figure 5, it was amazing that the identified 31 ingredients covered 30 flavones (22 flavonoids, 8 isoflavones) and 1 benzopyran, hinting that the classes of ingredients were simple, and flavones may be the primary bio-active substances in MOF and MEF. In addition, the major identified chemical components showed various distinct activities. Next, four representative constituents, including Quercetin, Biochanin A, Calycosin and Prim-O-glucosylcimifugin, were chosen for detailed bio-active explanation.

Quercetin is a polyphenolic flavonoid compound which is abundant in many kinds of flowers, leaves and fruits among a good deal of plants, as well as Chinese herbs. Current studies show that Quercetin plays a positive role in multiple diseases, especially such as tumors, cardiovascular diseases, osteoporosis and lung damage. Recent pharmacology studies have shown that Quercetin exhibited various activities in animals and cells, such as anti-oxidant [19,20], anti-inflammatory [21], anti-bacterial [22] and anti-atherosclerosis [23] activities. When talking about the compound named Biochanin A, a vital representative O-methylated isoflavone ingredient, it is a phytoestrogen largely produced during the growth of different kinds of legume plants such as chickpea, red clover and “Huangqi”. Biochanin A has great development prospects in health products and natural medicines based on its multiple biological functions such as anti-tumor [24,25], anti-inflammatory [26], anti-viral [27] and neuroprotection action [28]. In addition to this, another O-methylated isoflavonoid compound named Calycosin was successfully screened out in MOF and MEF. It is worth noting that Calycosin was deemed as the representational flavonoid compound for quality standard of “Huangqi” in Chinese pharmacopoeia (2020). The content of Calycosin could evaluate and control the quality of “Huangqi” derived from disparate sources (MO and ME), multifarious producing areas and different growing years, indicating the important role of Calycosin in the “Huangqi” herb. According to the present results, to our surprise, Calycosin was located for discriminating MOF and MEF on account of its different content. In recent years, modern pharmacological studies of Calycosin have been conducted in depth. Many articles have demonstrated that Calycosin possesses multiple activities, such as anti-tumor [29], anti-oxidant [30,31], heart and angiocarpy protection [32,33], lung protection [34], neuroprotection [35,36], anti-liver fibrosis [37], anti-hepatic steatosis [38], anti-inflammatory [39,40] and anti-diabetic kidney disease [41,42] activities, hinting that Calycosin may be a bio-active constituent in MOF and MEF. Moreover, Prim-O-glucosylcimifugin is a characteristic benzopyran compound identified in MOF and MEF with multifold activities. Wanfeng Gao [43] studied the anti-tumor effect of PD-1 by targeting myeloid-derived suppressor cells. The results of this study showed that Prim-O-glucosylcimifugin bonded well with the target proteins, inhibited the arginine metabolism and tricarboxylic acid cycle, increased CD8 T-lymphocyte infiltration in tumors and enhanced the effect of PD-1 inhibitor in B16-F10 and 4 T1 mouse tumor models. The results provided a potential option for promoting the efficacy of PD-1 inhibitors in clinical practice. Ouyang Ping [44] proved that Prim-O-glucosylcimifugin could protect A549 cells from Hla-medicated injury, showing great capacity in treating *S. aureus* infections against Hla. Yu Yin [45] studied the protective effect of Prim-O-Glucosylcimifugin on ulcerative colitis in vitro and in vivo and revealed that Prim-O-glucosylcimifugin could repair the integrity of the intestinal barrier and regulate the diversity and abundance of intestinal flora against the ulcerative colitis through inhibiting the activation of MAPK, AKT and NF-κB signaling pathways. Beyond that, Prim-O-glucosylcimifugin also showed a positive role in anti-nociception action by downregulating spinal COX-2 expression [46], and exhibited scavenging activities of DPPH anion and ABTS cation radicals [47]. Summing up the above, according to a large number of studies, the screened flavonoid metabolites between MOF and MEF, in particular Quercetin, Biochanin A, Calycosin and Prim-O-glucosylcimifugin, possess manifold bio-activities, indicating that the pharmacological and health protection actions of MOF and MEF may be quite different.

**Figure 6:**
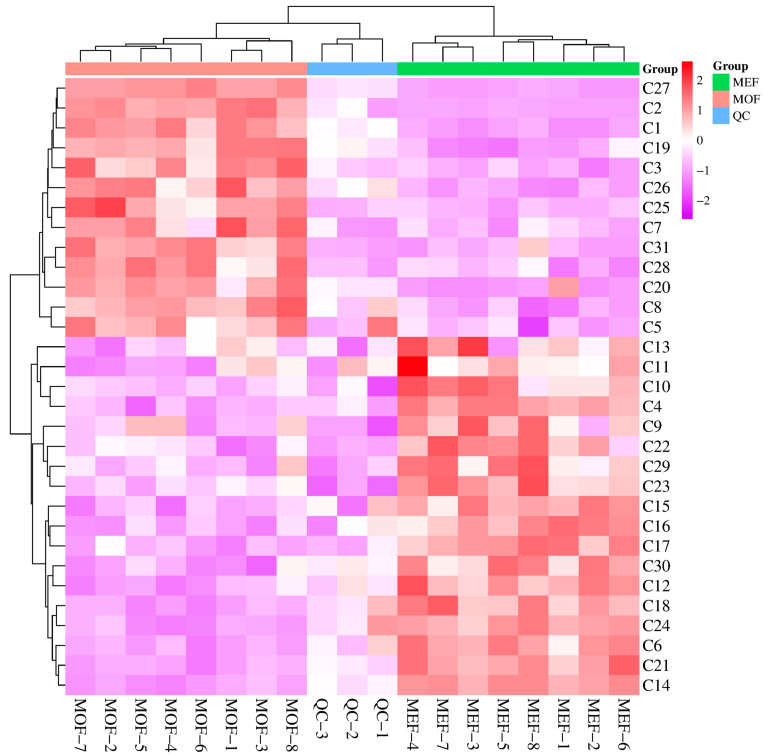
The heat map of ions between MOF and MEF using the UHPLC−Q−TOF−MS/MS approach.

As MOF and MEF originated from different sources of “Huangqi”, MOF and MEF may possess diverse secondary metabolic profiles. The metabolomic results showed that the chemical biomarkers between MOF and MEF are mainly composed of flavonoids, terpenoids and phenolic acids. Metabolomics is a high-throughput means which can rapidly analyze the different components in different plants and clarify the primary and secondary metabolites on different levels. The results obtained from the metabolomic method could provide a reliable basis for the quality control of Chinese herbs. On the basis of the metabolomic multivariate analysis between MOF and MEF, a heat map (Figure 6) was ultimately established to display the contents of chemical metabolites in order to comprehend the relative intensity among diverse components in MOF and MEF. In Figure 6, relative comparisons among ingredients between MOF and MEF were conducted to uncover the chemical markers directly. As displayed in Figure 6, 18 components which had obvious higher contents in MEF than in MOF were screened out, including **C4**, **C6**, **C9**, **C10**, **C11**, **C12**, **C13**, **C14**, **C15**, **C16**, **C17**, **C18**, **C21**, **C22**, **C23**, **C24**, **C29** and **C30**. However, 13 other ingredients showing lower contents in MEF than in MOF were screened out, including **C1**, **C2**, **C3**, **C5**, **C7**, **C8**, **C19**, **C20**, **C25**, **C26**, **C27**, **C28** and **C31**. The present results hint that MEF may possess more activities and wider applications than MOF.

For plants, secondary metabolites are particularly important for the development of growth, and the production and distribution of chemical ingredients are specific in different species, organs, tissues and stages; however, the kinds of secondary metabolites in different parts of the same plant may be similar. Hence, the chemical understanding of MOF and MEF is extremely meaningful. From the above results of MEF and MOF, flavonoid ingredients with diverse activities were abundant, indicating that flavonoid ingredients were the foremost secondary metabolites in MOF and MEF. In recent years, with the exploitation of the by-products of TCM, as well as the awareness of healthcare, the study of by-products has been paid close attention by researchers. Furthermore, with the development of new drugs from natural medicine with low toxicity/cost and high content/activity, MOF and MEF may shows broad development prospects in the field of TCM.

## 3. Materials and Methods

### 3.1. Materials and Chemicals

MOF and MEF were collected from the medical plants garden of Qiqihar Medical University and identified by professor Jicheng Liu from Qiqihar Medical University. The 0.22 μm filter membranes were purchased from Tianjin Jinteng experimental equipment Co., Ltd. (Tianjin, China). Methanol, acetonitrile and formic acid were of HPLC-grade. Methanol and acetonitrile were obtained from Merck company (Darmstadt, Germany), and formic acid was obtained from Thermo Fisher Scientific (Pittsburgh, PA, USA). Experimental distilled water was depurated alodial water obtained via a Milli-Q pure-ultrapure system (Millipore, Bedford, MA, USA). In addition, other required chemicals were of analytical grades.

### 3.2. Extraction of Samples for UHPLC−Q−TOF−MS/MS Analysis

The obtained MOF and MEF were dried in a drying oven under 50 °C for 4 h. Then, the dried MOF and MEF were smashed and filtered through a No. 4 sieve mesh. Eight samples of MOF and MEF powder weighing 1 g were taken out and extracted with ultrasound-assisted extraction for 30 min with 40 mL of 70% methanol, separately. An isopycnic of all the extracted solutions was mixed as a QC sample. Then, all the proper amounts of supernatants were obtained and filtered through a 0.22 μm membrane. Finally, 3 μL MOF and MEF sample solutions were detected using an UHPLC−Q−TOF−MS/MS system.

### 3.3. Conditions of UHPLC−Q−TOF−MS/MS Platform

In the present analysis, an ultra-high performance liquid chromatography system named LC-30A (Shimadzu Corporation, Kyoto, Japan) combined with a mass spectrometer named Triple TOF 4600 (AB Sciex Corporation, Redwood city, CA, USA) was utilized for data acquisition. The optimized parameters of liquid chromatography and mass spectrometry are shown as follows.

Liquid chromatography conditions: An ultra-efficient separation column known as ACQUITY^TM^ UPLC HSS T_3_ column (100 mm × 2.1 mm i.d., 1.8 μm, Waters Corporation, Milford, MA, USA) was adopted for chromatographic separation. The column temperature was maintained at 40 °C during sample analysis. Water containing 0.1% formic acid (A) and acetonitrile containing 0.1% formic acid (B) were selected as the optimal mobile phases. The following gradient elution program was installed: 0.01–2.5 min, 5–25% B; 2.5–3.5 min, 25–50% B; 3.5–4.5 min, 50–75% B; 4.5–7.5 min, 75–75% B; 7.5–8 min, 75–100% B; 8–15 min, 100–100% B. The flow velocity of mobile phase was maintained at 0.4 mL/min, and the injection volume of the sample solution was 5 μL. Under these conditions, both the quantity and quality of detected chromatographic peaks of MOF and MEF samples were satisfactory.

Mass spectrum conditions: Electrospray ionization was employed in the mass spectrometry system, and the temperature was kept at 600 °C. Two classic acquisition modes, including POS and NEG ion modes, were conducted several times in order to collect full-scale data. Under POS acquisition course, the parameter of ionspray voltage floating (ISVF) was maintained at 5500 V, and pressures of nebulizer gas, auxiliary gas and curtain gas were, respectively, held at 55 psi, 55 psi and 30 psi. Moreover, voltages of declustering potential and collision energy (CE) were set as 100 V and 10 V. In order to obtain befitting primary fragments, the TOFMS acquisition period was accomplished in 0.15 s. Under the NEG acquisition process, ISVF was kept at −4000 V, and other conditions were the same as in the POS source. In the data acquisition stage, the idiomatical information-dependent acquisition (IDA) mode was selected to synchronously collect MS and MS/MS information in one period. When referring the parameters of IDA experiment, the CE was between 20 and 60 V, and 10 nominated ions were supervised per IDA acquisition period. Furthermore, a dynamic background subtract mode which intelligently distinguished the background or matrix MS/MS ions from other endogenous or exogenous components was used. During the time of sample acquisition, the UHPLC−Q−TOF−MS/MS system was calibrated in two hours.

### 3.4. Matrix Establishment and PCA Analysis

The original data files (.wiff and wiff.scan) were imported into the MarkerView 1.2 software for peak extraction, peak matching and peak alignment operations. After processing, a matrix covering rich information like retention time, *m*/*z* and peak area were obtained. Then, grouping was conducted according to sample attributes, and normalization processing was carried out after isotopes were removed. Finally, PCA was performed to characterize the clustering information between the MOF group and the MEF group. Then, the matrix was further imported into Ezinfo3.0 software for OPLS-DA analysis, and the statistical analysis was conducted between the MOF group and the MEF group.

### 3.5. Screening and Identification of Chemical Markers

Ions whose *p* value was less than 0.05 and had a VIP greater than 1.0 were screened out; then, the information including retention time and *m*/*z* were, respectively, exported to PeakView 2.2 plug-in MasterView 1.1 software. Furthermore, the standard database (HR-MSMS-Spectral-Library) was used for non-target research. Finally, compounds with a mass error less than 10 ppm and MS/MS matching score greater than 70 were deemed as the identification of chemical markers.

## 4. Conclusions

In the present paper, the metabolomics method was successfully applied to differentiate the chemical ingredients between MOF and MEF. With the reasonable combination of rapid UHPLC−Q−TOF−MS/MS technology and visual data analysis methods, the MOF and MEF were visibly separated into two scopes in PCA loading plots. In total, 31 differential components between MOF and MEF were located and successfully identified; among them, 30 were flavonoid ingredients, meaning that flavonoids may be the dominating bio-active members. Meanwhile, 18 active ingredients, particularly Calycosin, Cyanidin-3-O-glucoside, Quercetin, Rutin, Kaempferol, Formononetin, Isomucronulatol and Prim-O-glucosylcimifugin, had higher contents in MEF than in MOF. However, 13 ingredients, such as Biochanin A, Tectoridin, Isomucronulatol-7-O-glucoside, Liquiritin and Rhamnetin, had lower contents in MOF than in MEF. It is worth noting that most of the chemical markers with higher levels in MEF exhibited plentiful activities, especially anti-oxidant, anti-inflammatory, anti-bacterial and anti-tumor ones, indicating that MEF may have wider applications in the medical and health fields. In other words, significant differences in chemical ingredients between MOF and MEF were found, and flavonoids played a critical role in this chemical discrepancy. The obtained data offered useful information for the classification and identification between MOF and MEF, providing an explicit reference point for further extraction and enrichment studies of flavonoid constituents in MOF and MEF. The results elucidated the chemical labels between MOF and MEF on the whole, contributing to the deep exploitation of TCM’s accessory substances. In addition, the established method was efficient and rapid and could be adapted for the chemical understanding of other Chinese herbs.

## Figures and Tables

**Table 1:** The information of the identified chemical metabolites for MOF and MEF under the POS/NEG ion mode using UHPLC−Q−TOF−MS/MS.

No.	Retention Time (min.)	*m*/*z*	CAS Numbers	Identified Results	Adducts	Formulas	Mass Error (ppm)
**C1**	2.76	417.1210	551-15-5	Liquiritin	M − H	C_21_H_22_O_9_	4.64
**C2**	2.94	445.1503	94367-43-8	Isomucronulatol-7-O-glucoside	M − H_2_O − H, M + FA − H	C_23_H_28_O_10_	−0.29
**C3**	3.55	625.1463	99224-12-1	Herbacetin-3,8-diglucopyranoside	M − H, M + FA − H	C_27_H_30_O_17_	8.36
**C4**	3.64	343.0848	41365-32-6	4′,5-Dihydroxy-3′,6,7-trimethoxyflavone	M − H, M + FA − H	C_18_H_16_O_7_	7.20
**C5**	3.73	609.1512	52187-80-1	Luteolin-7,3′-di-O-glucoside	M − H	C_27_H_30_O_16_	8.30
**C6**	4.04	449.1116	210050-28-5	Hovetrichoside C	M − H	C_21_H_22_O_11_	5.86
**C7**	4.08	625.1459	29125-80-2	Quercetin 3,4′-diglucoside	M − H, M + FA − H	C_27_H_30_O_17_	7.86
**C8**	4.21	479.0868	15648-86-9	Myricetin 3-galactopyranoside	M − H	C_21_H_20_O_13_	7.63
**C9**	4.30	609.1515	153-18-4	Rutin	M − H	C_27_H_30_O_16_	8.87
**C10**	4.46	593.1558	17353-03-6	Kaempferol-7-O-neohesperidoside	M − H	C_27_H_30_O_15_	7.68
**C11**	4.48	287.0541	520-18-3	Kaempferol	M + H	C_15_H_10_O_6_	−3.17
**C12**	4.50	449.1067	23627-87-4	Kaempferol 3-ss-D-galactoside	M + H	C_21_H_20_O_11_	−2.57
**C13**	4.63	551.1016	96862-01-0	6″-O-Malonylisoquercitrin	M + H, M + Na	C_24_H_22_O_15_	−2.82
**C14**	4.68	519.1125	51011-05-3	6″-O-Malonylgenistin	M + H	C_24_H_22_O_13_	−1.51
**C15**	4.74	449.1065	7084-24-4	Cyanidin-3-O-glucoside	M + H	C_21_H_20_O_11_	−2.95
**C16**	4.77	479.1169	5041-82-7	Isorhamnetin 3-O-glucoside	M + H	C_22_H_22_O_12_	−3.19
**C17**	4.77	317.0645	603-61-2	Tamarixetin	M + H	C_16_H_12_O_7_	−3.30
**C18**	5.12	513.1653	80681-45-4	Prim-O-glucosylcimifugin	M + FA − H	C_22_H_28_O_11_	8.38
**C19**	5.14	317.0646	90-19-7	Rhamnetin	M + H	C_16_H_12_O_7_	−3.03
**C20**	5.15	515.1234	77307-50-7	Kaempferol 3-O-(3″,4″-di-O-acetyl-α-L-rhamnopyranoside)	M − H	C_25_H_24_O_12_	7.55
**C21**	5.15	533.1278	137705-39-6	6″-O-Malonylglycitin	M + H	C_25_H_24_O_13_	−2.24
**C22**	5.21	301.0364	117-39-5	Quercetin	M − H	C_15_H_10_O_7_	3.55
**C23**	5.21	299.0210	90-18-6	Quercetagetin	M − H_2_O − H, M − H	C_15_H_10_O_8_	3.89
**C24**	5.24	285.0747	20575-57-9	Calycosin	M + H	C_16_H_12_O_5_	−3.55
**C25**	5.24	301.0697	491-54-3	3,5,7-Trihydroxy-4′-methoxyflavone	M + H	C_16_H_12_O_6_	−3.08
**C26**	5.24	463.1224	20126-59-4	Diosmetin-7-O-β-D-glucopyranoside	M + H, M + Na	C_22_H_22_O_11_	−2.27
**C27**	5.25	461.1120	611-40-5	Tectoridin	M − H	C_22_H_22_O_11_	6.63
**C28**	5.50	299.0572	20243-59-8	Luteolin 7-methyl ether	M − H	C_16_H_12_O_6_	3.70
**C29**	5.66	267.0674	485-72-3	Formononetin	M − H	C_16_H_12_O_4_	4.34
**C30**	5.76	301.1095	64474-51-7	Isomucronulatol	M − H	C_17_H_18_O_5_	4.35
**C31**	5.97	283.0626	491-80-5	Biochanin A	M − H	C_16_H_12_O_5_	4.87

## Data Availability

The original contributions are included in the article, and further inquiries can be obtained upon request to the corresponding author.
